# MSCs: Scientific Support for Multiple Therapies

**DOI:** 10.1155/2015/280572

**Published:** 2015-07-29

**Authors:** Mark F. Pittenger, Katarina Le Blanc, Donald G. Phinney, Jerry K. Y. Chan

**Affiliations:** ^1^Department of Surgery, University of Maryland School of Medicine, Baltimore, USA; ^2^Department of Medicine, Division of Clinical Immunology, Karolinska Institute, Stockholm, Sweden; ^3^Department of Molecular Therapeutics, The Scripps Research Institute-Florida, USA; ^4^Department of Reproductive Medicine, K.K. Women's and Children's Hospital, Yong Loo Lin School of Medicine, NUS/Duke-National University Hospital, Singapore

Whether we call them Mesenchymal Stem Cells, Mesenchymal Stromal Cells, Multipotent Stromal Cells, Skeletal Stem Cells, or another name, this class of stem/progenitor cells has been continuously studied for more than 20 years. Over 30,000 papers have been published on MSCs and over 10,000 patients have been administered MSC or MSC-like cells in over 300 clinical trials. Importantly, the first autologous* in vitro* cultured MSCs were injected into recipients over two decades ago, and the safety record for MSCs remains strong. Nevertheless, there remains much to learn about MSC science and their therapeutic potential. For example, the varied approaches taken by different laboratories to exploit the MSC therapeutic potential speaks to the complexity of MSCs and the ingenuity of those that study the cells. One size does not fit all.

The ease of isolation and propagation of MSCs means that anyone can become proficient in culturing MSCs but there is a learning curve for MSC assay reproducibility. However, once this proficiency is achieved new results can be produced with confidence. The field continues to seek new assays and metrics that reliably and reproducibly predict the therapeutic efficacy of a MSC preparation. Also, a typical clinical dose of MSCs is 100 million cells in 30–50 mL, which is approximately 400 uL of packed cells. Therefore, a 70 kg patient is typically administered 0.5 mL of adult stem cells but once injected this dose of cells diminishes quickly such that within ~48 hrs only 10% may be left, further diminishing to 1-2% over the next 48 hrs. Therefore, it is quite remarkable that the administered MSCs have any therapeutic effect. Consequently, it is critical to further improve our measurement of the survival of transplanted cells and their biodistribution to better predict their clinical efficacy and potency.

In this issue, seven MSC research groups present new work or focused reviews on MSCs.

The acknowledged founder of the MSC field and one of its strongest advocates, A. I. Caplan, has thought carefully about MSCs for many decades and therefore his perspectives on the field at large are always informative. Bone repair played a major role in the discovery and initial studies of MSCs and bone tissue serves as a clear example of where MSCs participate and persist in the healed tissue. Here, A. I. Caplan reminds us that MSCs or MSC-like cells are found in all tissues and provide local control over inflammation and tissue repair by facilitating the actions of resident cells and modifying the milieu of factors at sites of repair and regeneration.

Over 300 MSC-based clinical trials have been registered at http://clinicaltrials.gov/. As N. Escacena et al. point out the large number of clinical studies reported have varied in their efficacy and outcomes, demonstrating that we still have ways to go to use MSCs effectively across many therapeutic areas. Knowing more about the patients to be treated as well as the tissue source and method of MSC preparation and their therapeutic administration should improve the overall results of clinical studies. While this is clearly desirable, the early death of administered MSCs and its effects on the patient's physiology need careful consideration. At this juncture, MSCs are used primarily for their cytokine and growth factor production rather than for their cell replacement and differentiation ability. There are several reasons for this approach including the following: (1) MSCs secrete powerful anti-inflammatory factors and all tissue injury is accompanied by inflammation; (2) MSCs produce angiogenic factors and interaction with other cell types further enhances their production of these factors, which are needed during tissue repair; (3) MSCs become adapted to* in vitro* metabolic conditions; and (4) most MSCs do not survive the quick transition to* in vivo* conditions. Therefore, the field still needs reliable measures of cell survival and engraftment both short term and long term following administration to patients.

S.-Y. Kao et al. isolated MSCs from pancreatic tissue as well as bone marrow and Wharton's jelly and studied their differentiation into insulin producing cells. These authors have previously generated insulin producing cells to treat diabetic mice and rats and this study tested the effects of insulin producing MSCs from different sources in the streptozotocin-induced diabetic rats.

M. Ferretti et al. provide a study of the interaction of chondrocytes isolated from osteoarthritic patients with their bone marrow derived MSCs. The interaction of the two cell types provides much more production of hyaluronan, an important player in joint healing. This is a theme that persists in cell therapy but remains underappreciated, in which cell-cell interactions provide a dynamic environment not evident when single cell types are studied or applied to damaged tissue. As we develop expertise in single cell therapy, intentional combination cell therapies will need to be carefully tested.

Low level laser therapy has been available clinically for several decades and has been used to stimulate wound healing and hair growth. Mechanistically, laser light can increase collagen synthesis or stimulate IL-8 and IL-1*α* production. T. Kushibiki et al. provide a study of laser light effects on MSCs and osteogenic differentiation. Laser light provides another tool that many investigators are not familiar with to stimulate dynamic changes in MSCs.

For several years, W. C. W. Chen et al. have studied the multipotent cells available in the vasculature associated with the intima, media, and adventitia layers of vessels, collectively known as microvascular pericytes. The culture of these pericytes results in MSC-like cells when various assays are performed. The availability of these reparative cells along all vessels raises the possibility of immediately available* in situ* regenerative cells when injury occurs. Here the authors review the characterization and regenerative potential of pericytes. As tissue injury healing slows with age, the question remains why bone marrow MSCs and microvascular pericytes work so well for tissue healing when we are young, but not after we grow old.

The case has been made in the past that MSCs may promote tumor growth due to their trophic effects and production of angiogenic factors. MSCs have a propensity to migrate to wounds and damaged tissue, and tumors are characterized as wounds that do not heal. With this in mind, gene modified MSCs have been tested for their ability to deliver anticancer compounds in experimental models. Past work in this area has shown that MSCs isolated from patients with myeloproliferative disorders do not have the identified oncogenic JAK2 mutation suggesting that MSCs and the tumorigenic hematologic progenitor cells did not share a mutated common ancestor. As MSCs migrate to tumors, other researchers have used them to deliver interferon *β* to tumors. In this issue P. Johann and I. Müller review the evidence that MSCs may aid solid tumor growth and compare them with tumor associated fibroblasts that have a longer literature history than the more recently described MSC.

*Mark F. Pittenger* *Mark F. Pittenger* *Katarina Le Blanc* *Katarina Le Blanc* *Donald G. Phinney* *Donald G. Phinney* *Jerry K. Y. Chan* *Jerry K. Y. Chan*

